# Therapeutic potential of Golta therapy via radiographic and thermographic evaluation in improving scoliosis: A case report

**DOI:** 10.1097/MD.0000000000050830

**Published:** 2026-09-25

**Authors:** Hong-Seok Yoo, Sung-Hoon Kim

**Affiliations:** a Bonecare Korean Medicine Clinic, Seoul, Republic of Korea; b College of Korean Medicine, Kyung Hee University, Seoul, Republic of Korea.

**Keywords:** adolescent idiopathic scoliosis, Cobb angle, digital infrared thermography, Golta therapy, manual therapy

## Abstract

**Rationale:**

Golta therapy is a manual spinal correction technique using percussive tapping instruments to mobilize the vertebrae and improve postural alignment. However, radiographic and thermographic changes following Golta therapy in patients with scoliosis have not been well documented.

**Patient concerns:**

A 13-year-old female presented with progressive postural asymmetry, including uneven shoulder height, an asymmetric waistline, and right-sided rib prominence.

**Diagnoses:**

Radiographic evaluation confirmed scoliosis, with baseline thoracic and lumbar Cobb angles of 38.26° and 34.00°, respectively. Thermographic evaluation demonstrated paraspinal temperature asymmetry.

**Interventions:**

The patient received 14 sessions of Golta therapy over 8 weeks, consisting of tapping-based vertebral and paraspinal mobilization.

**Outcomes:**

After treatment, the thoracic Cobb angle decreased from 38.26° to 30.54°, while the lumbar Cobb angle decreased from 34.00° to 20.00°. Thermographic evaluation showed reduced paraspinal temperature asymmetry, accompanied by improvement in postural symmetry. No adverse events were reported.

**Lessons:**

In this patient, Golta therapy was associated with improvements in radiographic Cobb angles, paraspinal thermal symmetry, and postural alignment without reported adverse events. Larger controlled studies with long-term follow-up are needed to determine the reproducibility, clinical significance, and durability of these findings.

## 1. Introduction

The human spine consists of 33 vertebrae subdivided into 5 regions: cervical (C1–C7), thoracic (T1–T12), lumbar (L1–L5), sacral (S1–S5), and coccygeal (Co1–Co4). It supports body weight and protects the spinal cord and nerves that innervate internal organs.^[1]^ Increasing evidence indicates that unfavorable conditions or injuries can lead to various spinal disorders, including degenerative diseases, deformities, trauma, and infections, often accompanied by pain, reduced mobility, and neurological complications.^[2]^

Scoliosis, lordosis, and kyphosis are the most common spinal deformities.^[3]^ Scoliosis is a complex 3-dimensional deformity characterized by both anteroposterior curvature and lateral deviation of the spine. Scoliosis is defined as a complex 3-dimensional deformity, characterized by both anteroposterior curvature and lateral deviation of the spine^[4]^ The condition often progresses during periods of rapid growth, particularly in puberty. Clinically, scoliosis is diagnosed using a Cobb angle of ≥10° and axial vertebral rotation.^[5]^ Approximately 80% of scoliosis cases are classified as adolescent idiopathic scoliosis (AIS), which affects individuals aged 11 to 18 years.^[6]^

Nonoperative treatments for scoliosis include pain management and physical therapy (e.g., gait training and core stabilization exercises),^[7]^ posture correction, and the use of orthotic braces.^[8,9]^ While surgery may be considered in severe cases of intractable pain, conservative treatment remains the 1st-line option. Bracing combined with exercise is primarily intended to slow down or prevent curve progression in AIS patients.^[10]^ However, nighttime bracing is generally effective only for curves ≤35,^[11]^ and scoliosis-specific exercises have shown benefits in mild scoliosis, although evidence of significant Cobb angle reduction is limited.^[12]^

Given these limitations, this study introduces Golta therapy as a nonoperative treatment approach with potential therapeutic benefits for scoliosis.

### 1.1. Therapeutic background of Golta therapy

Golta therapy is a newly developed therapeutic approach by Dr Hong-Seok Yoo based on the principle that spinal misalignment is closely linked to the onset and progression of various diseases. The spine not only provides mechanical support but also plays a crucial role in regulating the physiological and pathological functions of visceral organs.^[13]^ In traditional Korean medicine, therapeutic strategies emphasize spinal correction through bone tapping as a means of restoring balance and promoting systemic health. According to traditional acupuncture theory, back-shu (back-transporting) points are important acupuncture points located along the bladder meridian (BL) on the posterior aspect of the body, adjacent to the spine.

The governor vessel comprises 14 acupoints positioned at the inferior borders of the spinous processes. The 1st line of the BL included 20 points located 1.5 cun lateral to the spinous processes, whereas the 2nd line consisted of 14 points located 3 cun lateral (Fig. 1).^[14]^ Here, cun refers to a traditional body-proportional unit of measurement used in acupuncture, with its actual length varying according to the patient’s body dimensions.

**Figure 1.**
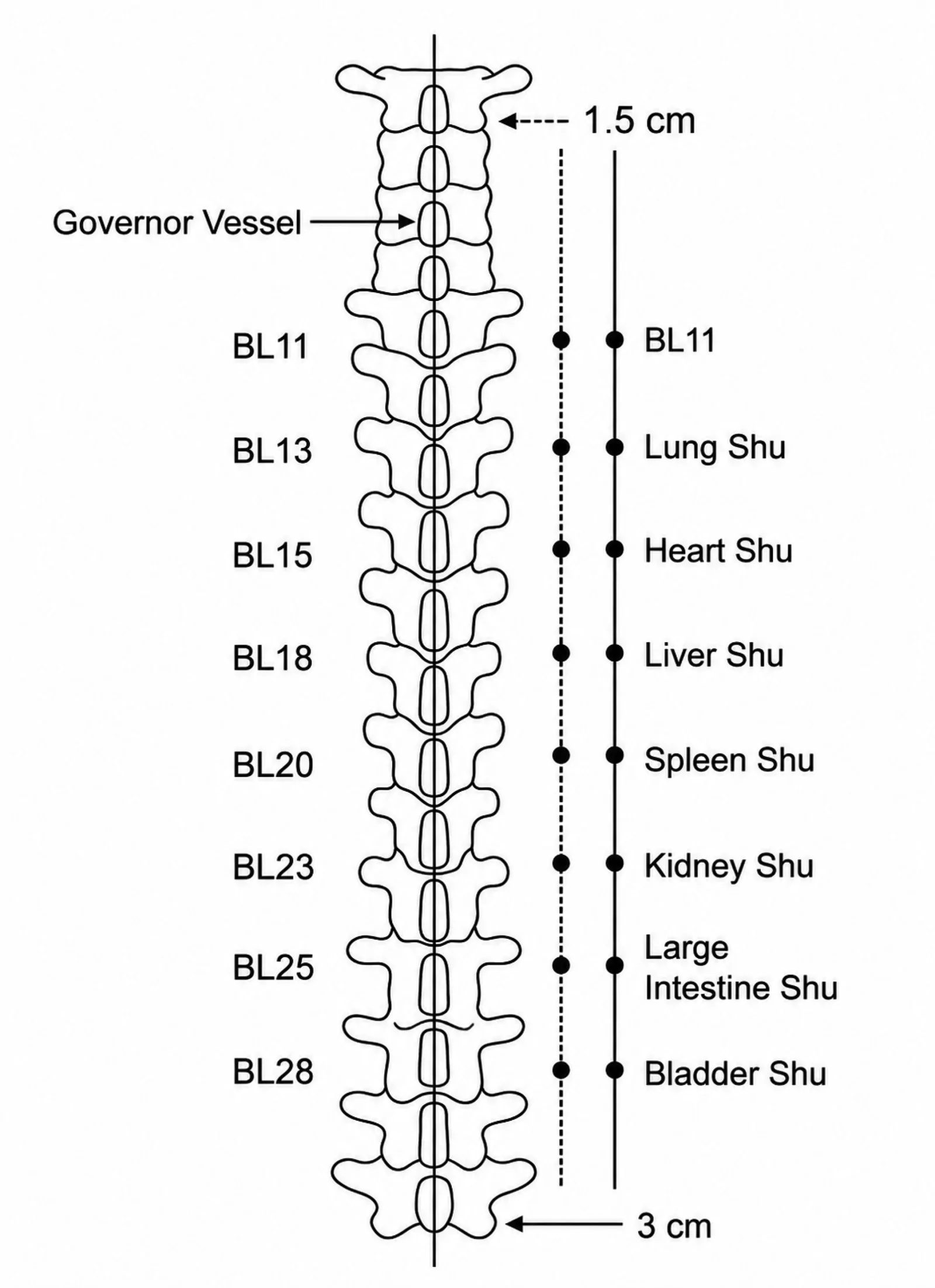
Anatomical distribution of the governor vessel and bladder meridian (BL) back-shu points in relation to the spinous processes. The governor vessel (Du Mai) runs along the posterior midline over the spinous processes. The bladder meridian runs bilaterally along 2 longitudinal lines on the back: an inner line located 1.5 cun lateral to the posterior midline and an outer line located 3 cun lateral. The back-shu points are located primarily on the inner bladder meridian line. Representative back-shu points include BL11, BL13 (lung shu), BL15 (heart shu), BL18 (liver shu), BL20 (spleen shu), BL23 (kidney shu), BL25 (large intestine shu), and BL28 (bladder shu).

From a modern anatomical perspective, these back-shu points correspond to regions innervated by the 4 major nerve plexuses of the spinal nerves that extend to the skin of the neck, back, waist, abdomen, and sacrum. These areas overlap with the same or neighboring spinal segments and nerve branches that supply the visceral organs.^[15]^ Based on this integrative understanding, Golta therapy posits that diseases such as intervertebral disc herniation, spinal stenosis, spinal deformities, gastrointestinal disorders, and even metabolic conditions such as diabetes mellitus may be ameliorated through correction of spinal misalignment via bone tapping. This is attributed to the interaction between internal organs and locomotor nerves through their connective tissues. Moreover, localized pain around the back-shu acupoints can be effectively alleviated by Golta therapy, further supporting its therapeutic value.

### 1.2. Tools of Golta therapy and their usages

Golta therapy employs 5 types of hammers and a single cylindrical bar as the primary instrument (Fig. 2A). As illustrated in Figure 2B, the treatment was typically performed by tapping the cylindrical bar either vertically or obliquely using a suitable hammer after identifying the tenderness points around the spinous processes.

**Figure 2.**
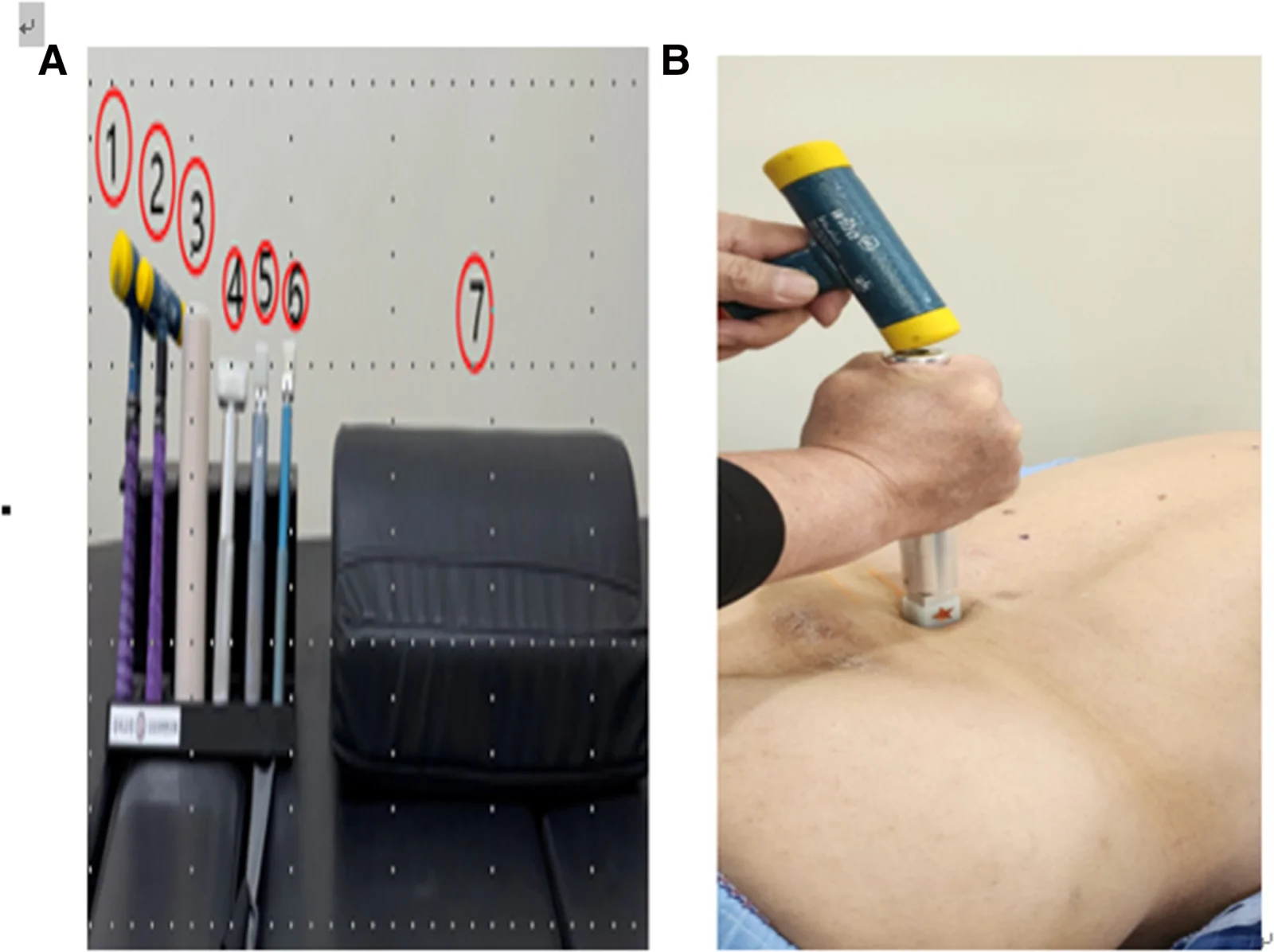
Tools of Golta therapy. (A) Six Golta tools: (1) Large hammer (used for generating strong percussive force, particularly for correcting anterior–posterior spinal deformities associated with bulging; requires more force than lateral correction). (2). Medium hammer (applied for moderate tapping to realign twisted or laterally deviated spines to the central axis). (3) Cylindrical bar (wooden intermediary bar, placed on pelvic or spinal points, commonly used for pelvic correction). (4) Thora bar (applied with the medium hammer for correction of lateral deviations in thoracic and lumbar vertebrae with prominent spinous processes). (5) Fron bar (used in the final correction stage after lateral adjustment, together with the large hammer, to address kyphotic deformities by pushing the spine inward). (6) Sacro bar (used with smaller spinous processes such as in cervical or sacral vertebrae, correcting lateral deviations). (7) Therapy mat (used to stabilize the patient during Golta therapy). (B) Picture for performing Golta therapy with a medium hammer and a cylindrical bar.

In cases of scoliosis, the cylindrical bar was positioned obliquely against the protruding spinous processes, and a large hammer was applied to deliver corrective tapping. This technique is intended to restore spinal balance by targeting the misaligned areas using controlled mechanical stimulation.

### 1.3. Safety considerations in Golta therapy

Even the most effective treatment becomes meaningless if it is not safe, as safety is the foremost priority in all medical practice. Regardless of favorable clinical outcomes, no therapy that carries the risk of severe or fatal side effects should be administered. Prior to administering Golta therapy, patients were thoroughly informed about its safety profiles. The procedure involved placing a cylindrical bar at specific points along the spine, which was then gently tapped with hammers to deliver controlled stimulation.

## 2. Case report

A 13-year-old Asian female residing in Anyang city and attending middle school presented with AIS. She had a history of scoliosis and had previously undergone 6 months of orthopedic management, including standard conservative treatment, before seeking Golta therapy. However, the previous treatment resulted in minimal improvement in postural asymmetry and no meaningful radiographic changes. She had no significant medical or surgical history or comorbidities. Her family history was negative for scoliosis, connective tissue disorders, and neuromuscular diseases. Psychosocially, she was otherwise healthy but reported cosmetic concerns and mild emotional stress related to her postural asymmetry. Genetic testing was not performed, and there were no clinical features suggestive of syndromic scoliosis.

The patient underwent 14 Golta therapy sessions between September 28 and November 27, 2013. Clinical evaluation included radiographic analysis, digital infrared thermal imaging (DITI), and computerized postural assessment performed before and after the intervention. Written informed consent was obtained from the patient’s legal guardian.

Golta therapy was performed using controlled percussive stimulation with a specialized intermediary tool and hammer. Percussive force was applied to the side of the vertebral or paraspinal region showing greater prominence, with the direction and intensity adjusted according to the patient’s spinal asymmetry and tolerance. The procedure was intended to provide mechanical corrective stimulation to the asymmetric spinal region while minimizing discomfort. Treatment intensity was individually adjusted at each session based on clinical observation and patient feedback.

The patient received 1 Golta therapy session during the 1st week, 2 sessions/wk during the following 6 weeks, and 1 session during the final week, for a total of 14 sessions over the 8-week treatment period. Tolerability was assessed through clinician observation and patient self-report at each visit. The patient demonstrated good adherence, reported no adverse effects, and tolerated all procedures without discomfort or treatment-related complications.

Spinal alignment was assessed using a standing posteroanterior whole-spine radiographic system (HNT Medical). Cobb angles were measured for both thoracic and lumbar curves using digital radiographic software (Thermal Imaging Analyzer). All radiographs were obtained under standardized positioning, and the measurements were independently verified by 2 clinicians to ensure accuracy and reproducibility. Baseline radiographic evaluation revealed a thoracic Cobb angle of 38.26° and a lumbar Cobb angle of 34°, consistent with moderate scoliosis on 2D spinal X-ray images in the posteroanterior view. Following 14 sessions of Golta therapy over the 8-week treatment period, the thoracic Cobb angle decreased from 38.26° to 30.54°, while the lumbar Cobb angle decreased from 34.00° to 20.00° (Fig. 3).

**Figure 3.**
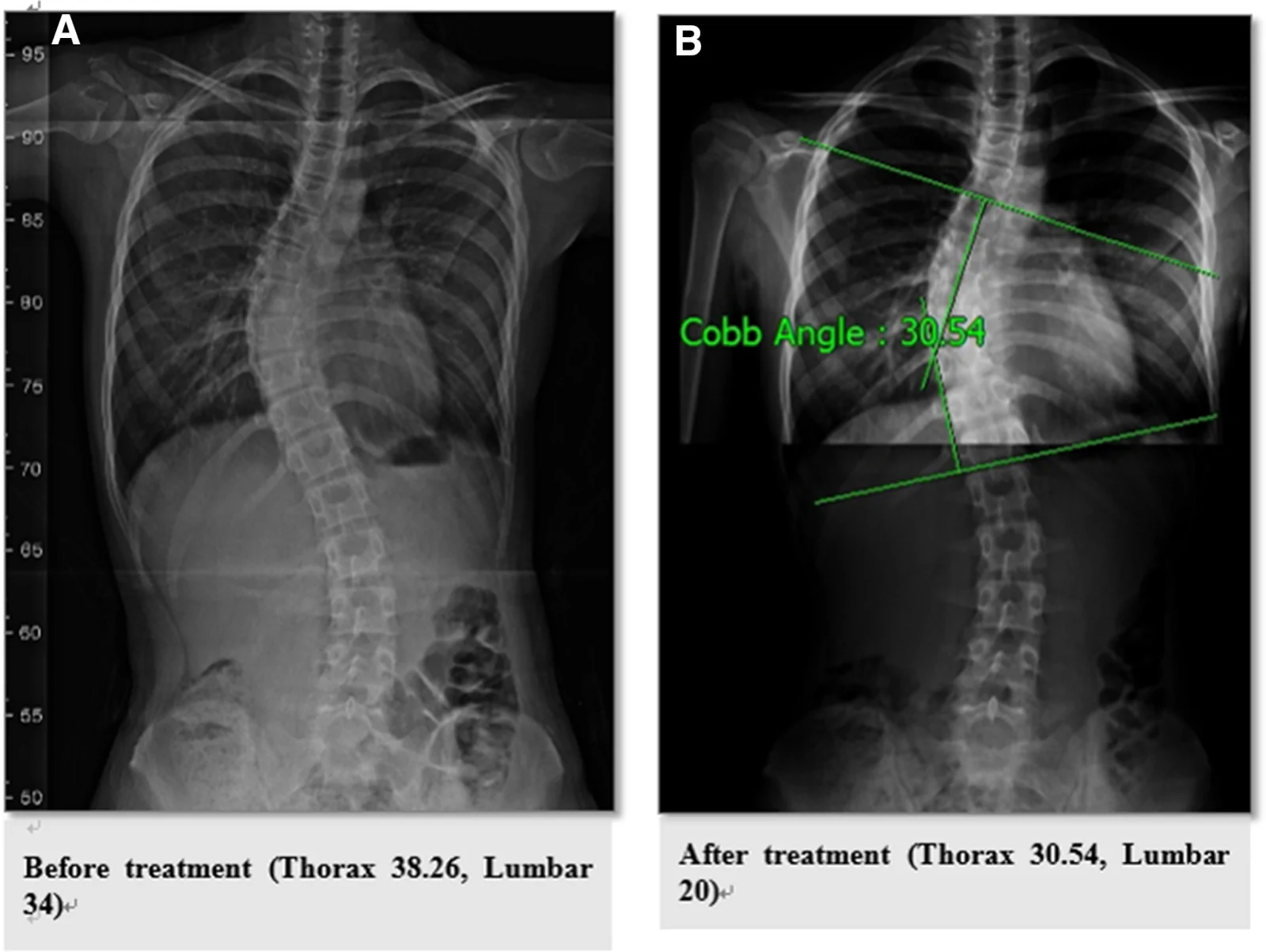
Radiographic changes before and after Golta therapy in a patient with scoliosis. (A) Before treatment: thoracic Cobb angle 38.26°, lumbar Cobb angle 34°. (B) After treatment: thoracic Cobb angle 30.54°, lumbar Cobb angle 20°, demonstrating marked improvement in spinal alignment.

The thermal distribution was evaluated using a digital infrared thermal imaging camera (MEDIKORS innerVue3). Prior to imaging, the patient was acclimatized for 15 minutes in a temperature-controlled room (22°C–24°C). Images were obtained in the standing posterior view, and thermal asymmetry was analyzed using the manufacturer’s dedicated software (DITI Visual Editor 3.0). Pre and posttreatment comparisons focused on changes in thermal symmetry and abnormally hot or cold regions. Before treatment, digital infrared thermal imaging demonstrated an irregular thermal distribution with asymmetry across the thoracolumbar region. After completion of Golta therapy, the thermal distribution appeared more balanced and symmetrical (Fig. 4).

**Figure 4.**
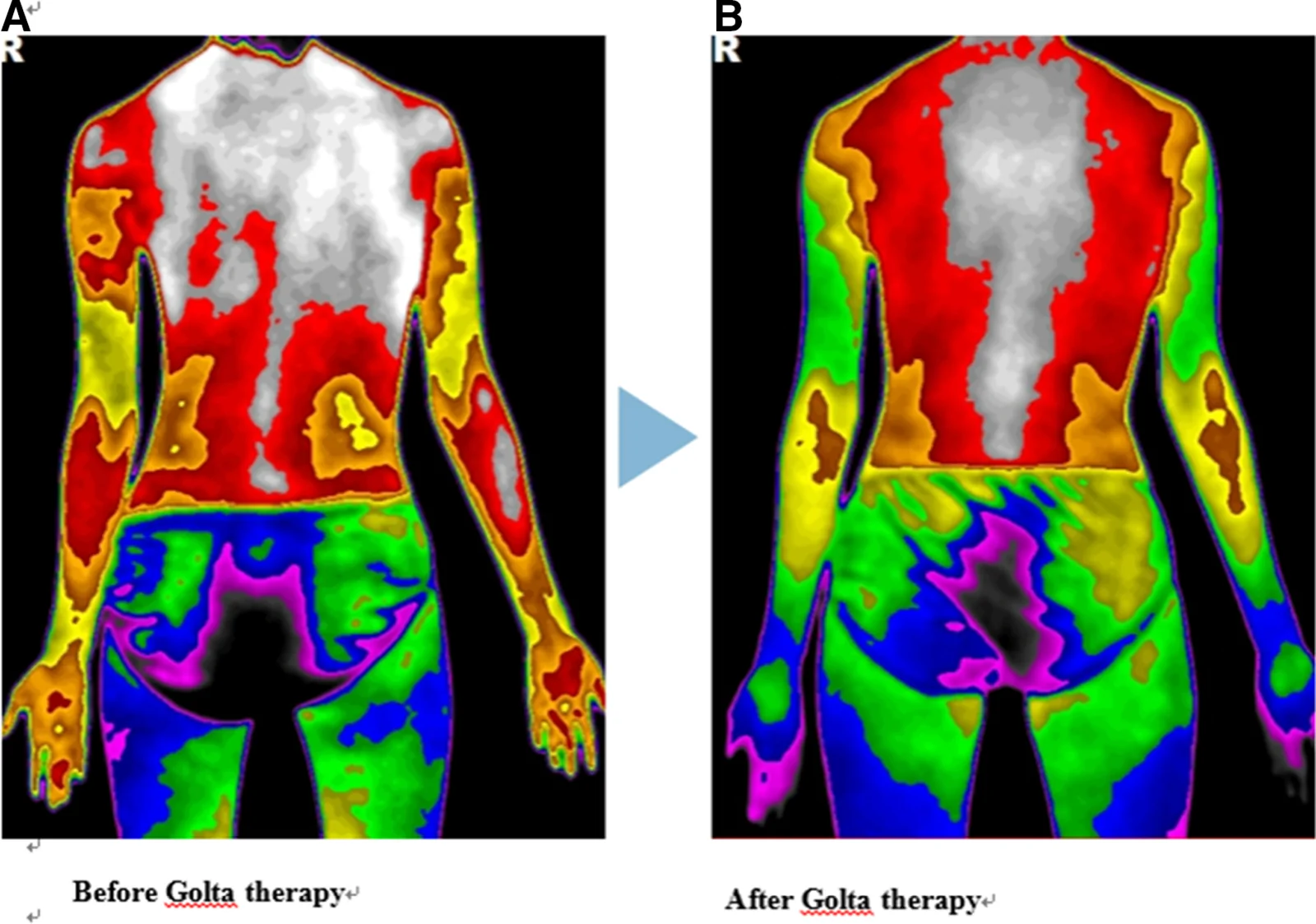
Thermographic assessment before and after Golta therapy using digital infrared thermal imaging. Thermographic images obtained with a digital infrared thermal diagnostic device show changes in heat distribution. (A) Before therapy, uneven and asymmetric thermal patterns are observed in the thoracolumbar region. (B) After therapy, thermal distribution appears more balanced and symmetrical, suggesting improved physiological condition.

Quantitative evaluation revealed shoulder angles of 33.97° and 22.68° before treatment, indicating marked asymmetry. After Golta therapy, the corresponding angles were 29.14° and 31.26°, showing greater bilateral symmetry. Improvements in pelvic alignment and trunk inclination were also observed, together with changes in overall postural balance (Fig. 5).

**Figure 5.**
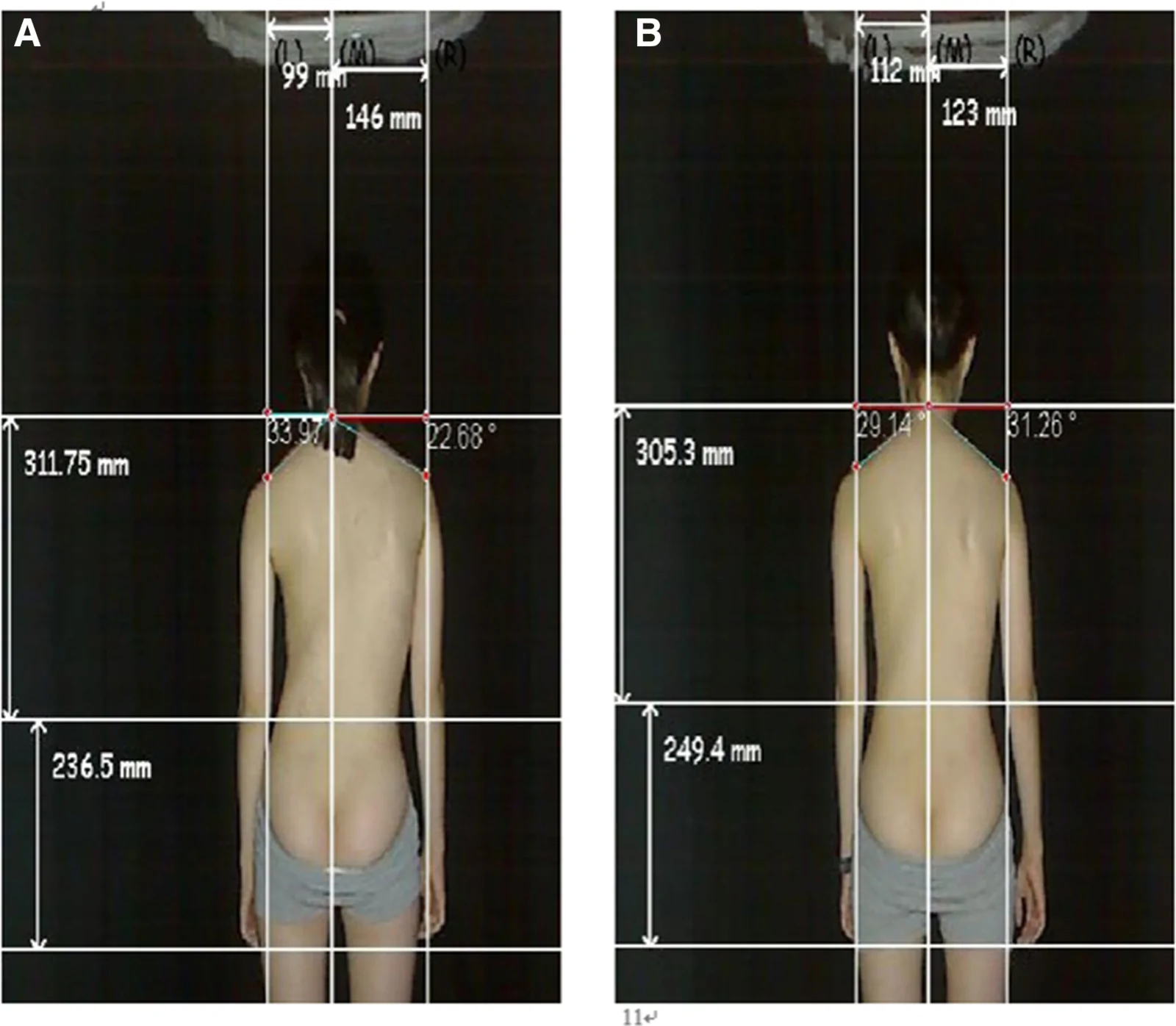
Postural assessment before and after Golta therapy. Photographic posture analysis using digital measurement tools shows changes in shoulder alignment and pelvic tilt. (A) Before treatment, postural asymmetry was observed, with shoulder-angle measurements of 33.97° and 22.68°, along with pelvic imbalance. (B) After treatment, the shoulder-angle measurements were 29.14° and 31.26°, accompanied by improved postural symmetry and pelvic alignment.

Regarding quality of life, the patient reported improved comfort, less discomfort related to postural asymmetry, greater satisfaction with her appearance, and increased confidence during daily activities and school participation. She also reported that the therapy was gentle and well tolerated.

## 3. Discussion

Although nonoperative interventions, including physical therapy, gait training, core stabilization exercises, postural correction, pain management, and orthotic bracing, have been used in the management of scoliosis,^[8,9]^ their effectiveness may vary according to disease severity, skeletal maturity, treatment adherence, and the type of intervention. In this context, the present case suggests the potential usefulness of Golta therapy as a complementary intervention for improving spinal alignment in an adolescent with scoliosis. After 14 treatment sessions over an 8-week period, radiographic and postural assessments demonstrated measurable changes. The Cobb angle is widely accepted as a standard radiographic measure for quantifying scoliosis severity by the Scoliosis Research Society.^[16]^ In the present case, reductions in both thoracic and lumbar Cobb angles indicated measurable improvement in spinal curvature following Golta therapy. These findings are consistent with previous reports suggesting that noninvasive manual or corrective interventions may contribute to changes in Cobb angle and postural alignment in adolescents with scoliosis.^[17–19]^

In addition to the radiographic changes, DITI analysis demonstrated a more symmetrical thermal distribution after treatment compared with baseline. Infrared thermography is a noninvasive technique that detects variations in skin surface temperature and has been used as a complementary tool for evaluating musculoskeletal dysfunction and monitoring treatment-related changes in spinal and other musculoskeletal disorders..^[20,21]^ In the present case, the observed improvement in thermal symmetry may reflect changes in physiological processes associated with the paraspinal region, including local circulation and muscle activity. Postural assessment also demonstrated reduced shoulder asymmetry, improved pelvic alignment, and better overall postural balance. Taken together, these multimodal findings suggest that Golta therapy may be associated with improvements in spinal alignment, musculoskeletal symmetry, and functional posture. However, because DITI provides an indirect physiological measure, the thermographic findings should be interpreted cautiously.

Although the findings of this case are encouraging, causality cannot be established from a single case report. The observed changes may have been influenced by patient-specific factors, natural variation, concurrent growth and development, or other unmeasured factors. In addition, long-term follow-up data were not available to determine whether the observed changes were maintained over time.

The combination of radiographic, thermographic, and postural assessments provided complementary information regarding structural and functional changes following treatment. Golta therapy involves controlled percussive stimulation delivered through intermediary tools. Similar manual and mechanical interventions, including spinal manipulation, vibration therapy, and traditional Korean medicine-based corrective approaches such as Chuna therapy, have been investigated for postural imbalance, degenerative spinal conditions, and chronic low back pain.^[22–24]^ Nevertheless, the mechanisms underlying Golta therapy remain unclear, and further experimental and clinical studies are required before its potential application to other spinal disorders can be determined.

To our knowledge, this is the 1st reported case investigating Golta therapy in adolescent idiopathic scoliosis. The use of multimodal evaluations, including Cobb angle measurement, DITI, and computerized postural assessment, allowed assessment of both structural and functional changes. However, this study has several limitations. As a single-patient case report, the generalizability of the findings is inherently limited. In addition, potential sex-related differences in spinal anatomy or treatment response were not evaluated. Previous studies have demonstrated sex-related variations in spinal dimensions and sagittal alignment, which may influence the clinical characteristics and management of spinal disorders.^[25]^

Furthermore, the present report focused specifically on adolescent idiopathic scoliosis and did not evaluate other etiologies of scoliosis. Congenital scoliosis, for example, may result from abnormal vertebral development, including incomplete formation of 1 side of a vertebral body, resulting in a hemivertebra.^[26]^ Other forms of scoliosis may be associated with degenerative, neuromuscular, traumatic, or structural abnormalities. Therefore, caution is required when extrapolating the findings of this case to patients with different types or causes of scoliosis.

Well-designed prospective studies involving larger and more diverse patient populations, standardized treatment protocols, appropriate comparison groups, and extended follow-up periods are needed to confirm the efficacy and safety of Golta therapy and to determine its potential clinical applicability in scoliosis and other spinal disorders.

## 4. Conclusion

Golta therapy resulted in notable radiographic and clinical improvements in this adolescent case of idiopathic scoliosis, suggesting its potential as a noninvasive therapeutic option. Although these findings were encouraging, they were derived from a single case report. Larger controlled studies with standardized protocols and long-term follow-up are necessary to confirm the clinical efficacy, safety, and durability of Golta therapy as a complementary or alternative management strategy for scoliosis.

## Acknowledgments

We sincerely thank the patient and her legal guardian for providing written informed consent for the publication of this case report.

## Author contributions

**Conceptualization:** Hong-Seok Yoo, Sung-Hoon Kim.

**Data curation:** Hong-Seok Yoo, Sung-Hoon Kim.

**Formal analysis:** Hong-Seok Yoo, Sung-Hoon Kim.

**Investigation:** Hong-Seok Yoo, Sung-Hoon Kim.

**Methodology:** Hong-Seok Yoo, Sung-Hoon Kim.

**Writing – original draft:** Hong-Seok Yoo, Sung-Hoon Kim.

**Writing – review & editing:** Hong-Seok Yoo, Sung-Hoon Kim.
